# Assembly of Dishevelled 3-based supermolecular complexes via phosphorylation and Axin

**DOI:** 10.1186/1750-2187-7-8

**Published:** 2012-06-29

**Authors:** Noriko Yokoyama, Nelli G Markova, Hsien-yu Wang, Craig C Malbon

**Affiliations:** 1 Departments of Pharmacology, School of Medicine, State University of New York at Stony Brook, Stony Brook, NY, 11794-8651, USA; 2 Departments of Physiology & Biophysics, School of Medicine, State University of New York at Stony Brook, Stony Brook, NY, 11794-8651, USA; 3 Department of Molecular Cell Physiology, Kyoto Prefectural University of Medicine, Kyoto, 602-8556, Japan

**Keywords:** Dishevelled, Oligomerization, Signalsomes, Phosphorylation, Supermolecular complexes, Wnt3a, Axin

## Abstract

**Background:**

Dishevelled-3 (Dvl3) is a multivalent scaffold essential to cell signaling in development. Dsh/Dvls enable a myriad of protein-protein interactions in Wnt signaling. In the canonical Wnt/β-catenin pathway specifically, Dvl3 polymerizes to form dynamic protein aggregates, so-called “signalsomes”, which propagate signals from the Wnt receptor Frizzled to downstream elements.

**Results:**

Very large Dvl3-based supermolecular complexes form in response to Wnt3a. These complexes are identified by steric-exclusion chromatography, affinity pull-downs, proteomics, and fluorescence correlation microscopy (*fcs*). In the current work, the roles of Dvl3 phosphorylation and of Axin in the assembly of Dvl3-based supermolecular complexes in response to Wnt3a are probed in totipotent mouse F9 teratocarcinoma cells. Point mutations of phosphorylation sites of Dvl3 which interfere with Lef/Tcf-sensitive transcriptional activation by Wnt3a are shown to interfere more proximally with the assembly of Dvl3-based supermolecular complexes. Axin, a Dvl-interacting protein, plays a central role in organizing the beta-catenin destruction complex. The assembly of Dvl3-based supermolecular complexes is blocked either by depletion of Axin or by mutation of Axin sites necessary for polymerization in response to Wnt3a.

**Conclusion:**

These data demonstrate that Wnt3a activation of the canonical pathway requires specific phosphorylation events as well as Axin to assemble very large, Dvl3-based supermolecular complexes; these complexes are a prerequisite to activation of Lef/Tcf-sensitive transcription.

## Background

Wnt/β-catenin signaling is mediated by Dishevelled (Dsh/Dvl) [1-3]. Binding of Wnt3a to Frizzled-1 (Fz1) and low-density lipoprotein receptor-related protein 5/6 (LRP5/6) facilitates the phosphorylation of LRP5/6, and triggers the interaction of Fz-LRP5/6 complex with dynamic multiprotein complexes. Dsh/Dvl is an essential scaffold protein, bridging the Wnt receptors to distinct downstream signaling components. Dvls oligomerize *via* their DIX domain and form dynamic protein assemblies (i.e., signalsomes) [4,5]. Expression of fluorescently-tagged fusion Dvl proteins displays large “punctate” structures [6-8]. Immunofluorescence images suggested that Wnt stimulates a formation of membrane-associated protein “aggregates/punctae” [9]. We have provided evidence that these punctae/aggregates are Dvl3-based supermolecular complexes, assembled during Wnt3a action. Dvl3-based supermolecular complexes assemble dynamically, are regulated temporally and spatially, and are necessary to enable Wnt3a activation of Lef/Tcf-sensitive transcription [10].

Mammalian Dvls include three isoforms, Dvl1, Dvl2 and Dvl3. Dvl3-deficient (−/−) mice die in the perinatal period due to profound developmental deficiencies [11]. In totipotent mouse F9 teratocarcinoma cells expressed Fz1, Wnt3a activates Lef/Tcf-sensitive transcription and primitive endoderm formation [12,13]; knockdown of Dvl3 blocks Wnt/β-catenin signaling [14]. Dvl isoforms display differential expression and localization [13,14]. The role of Dvl3, the least abundant isoforms, in supporting Wnt/β-catenin signaling is distinct from that of the far more abundant Dvl1 or Dvl2 [13]. Dvl3 plays a unique role in polymerizing dynamic multivalent, supermolecular complexes in a Wnt- and time-dependent manner [10].

Dvls are phosphoproteins, substrates for multiple kinases activated in response to Wnt. Upon Wnt stimulation Dvls show shifts of *M*_*r*_ on SDS-PAGE, characteristic of heavily phosphorylated proteins. A Wnt3a-induced shift of *M*_*r*_ of phospho-Dvls on SDS-PAGE has been shown to be dependent on the enzymatic activity of casein kinase (CK)1δ/ϵ [15,16]. CK1ϵ and CK1δ dock to Dvls and regulate Wnt signaling positively, actions presumed to reflect phosphorylation of Dvls [16-20]. CK1ϵ-dependent phosphorylation of Dvl, in turn, enhances the interaction of Dvl and Frat-1, preceding activation of Wnt/β-catenin signaling [19]. Although CK1δ/ϵ, CK2 and Par1 have been implicated as kinases that phosphorylate Dvls [15,21-23], a precise mapping of Wnt-dependent phosphorylation of Dvls is lacking. Phosphorylation is suspected to play critical roles in many Dvl protein-protein interactions. It has been shown that Dvl phosphorylation regulates protein docking, binding affinities, stability and trafficking of Dvls, as well as docking of other signaling elements essential to Wnt signaling [24].

Axin, like Dvls, is a scaffold protein essential to Wnt signaling. Axin contains two conserved domains, a RGS domain N-terminally and a DIX domain C-terminally. The RGS domain is the binding site of *adenomatous polyposis coli* (APC), whereas DIX domain mediates Axin homo-dimerization as well as docking of Dsh/Dvl [7,25-29]. A productive interaction between Axin and Dvls is obligate for Wnt-induced inhibition of the Axin destruction complexes [7,28,30]. In the destruction complexes, Axin acts as a scaffold for β-catenin, glycogen synthase kinase 3β **(**GSK3β), CK1 and APC, provoking the degradation of β-catenin [1,31,32]. In response to Wnt3a, β-catenin stabilizes, accumulates, and translocates to the nucleus, activating Lef/Tcf-sensitive transcription [2,3,33]. The cellular abundance of Axin is low. It has been argued on this basis that Axin may well be rate-limiting for the operation of the canonical Wnt pathway [34]. Whether Axin is an integral component of the Dvl3-based supermolecular complexes, however, has not been fully investigated.

Herein, we interrogate the assembly of dynamic, Dvl3-based supermolecular complexes in response to Wnt3a using size-exclusion chromatography, proteomic analysis and live cell imaging. Punctae/aggregates formed in response to Wnt3a appear to constitute Dvl3-based supermolecular complexes. The current study tests this hypothesis at the level of Dvl3 phosphorylation and of docking of Axin. Both Axin and specific sites of Dvl3 phosphorylation are shown to regulate assembly of Dvl3-based supermolecular complexes, as a prerequisite to activation of Lef/Tcf-sensitive transcription in response to Wnt3a.

## Results

### Dvl3 mutants defective in phosphorylation and polymerization block Wnt3a action

**Figure 1:**
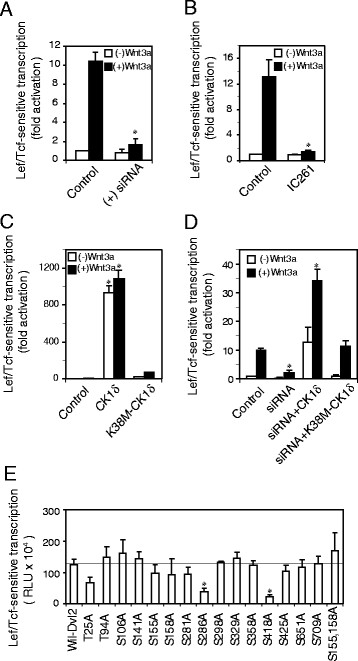
**CK1δ positively regulates Wnt/β-catenin signaling. ***Panel A, siRNA targeting CK1δ attenuates Lef/Tcf-sensitive transcription. *F9 cells were transfected with siRNA targeting CK1δ one day before co-transfection with Rfz1 and Super8xp TOPFlash (M50) reporter. On the following day, cells were either unstimulated or stimulated with Wnt3a for 7 hr. Cell lysates were assayed for Lef/Tcf-sensitive transcription. Results are displayed relative to the unstimulated cells (set to “1”). Results shown are mean values ± s.e.m. from 5 independent experiments. Statistical significance is indicated (*, *p *< 0.005). *Panel B, treatment of CK1δ ιnhibitor attenuates Lef/Tcf-sensitive transcription. *F9 cells were co-transfected with Rfz1 and M50. One day post transfection, cells were either untreated or pretreated with IC261 (20 μM, selective inhibitor of CK1δ/ϵ) for 1 hr prior to stimulation either with or without Wnt3a (20 ng/ml) for 7 hr in the continued presence (or absence) of IC261. Cell lysates were prepared and assay of Lef/Tcf-sensitive transcription was performed. Results are displayed relative to the unstimulated cells (set to “1”). Results shown are mean values ± s.e.m. from 3 independent experiments. Statistical significance is indicated (*, *p *< 0.005). *Panel C, expression of CK1δ enhances Lef/Tcf-sensitive transcription. *F9 cells were transfected with Rfz1, M50 and either wild-type CK1δ or K38M-CK1δ (kinase inactive mutant). On the following day, cells were either unstimulated or stimulated with Wnt3a for 7 hr. Cell lysates were assayed for Lef/Tcf-sensitive transcription. Results are displayed relative to the unstimulated cells (set to “1”). Statistical significance is provided, * denotes *p *< 0.005 (versus control cells). Results shown are mean values ± s.e.m. from 4 independent experiments. *Panel D, wild-type CK1δ rescues Lef/Tcf-sensitive transcription in the CK1δ-deficient cells. *F9 cells stably expressing Rfz1 and M50 were treated with siRNA targeting CK1δ one day before transfection with either wild-type CK1δ or K38M-CK1δ. On the following day, cells were either unstimulated or stimulated with Wnt3a for 7 hr. Cell lysates were assayed for Lef/Tcf-sensitive transcription. Results are displayed relative to the unstimulated cells (set to “1”). Statistical significance is provided, * denotes *p *< 0.005 (versus control cells). Results shown are mean values ± s.e.m. from 3 independent experiments. *Panel E, role of serine/threonine sites of Dvl2 in Lef/Tcf-sensitive transcription. *Phosphorylation of rDvl2 (purified from Sf9 cells) catalyzed by purified CK1δ was carried out *in vitro *and phosphorylation sites were identified by mass spectrometry*. *The phosphorylation sites (S/T) identified then were mutated to alanine using Quickchange Mutagenesis System (Stratagene, La Jolla, CA). F9 cell were co-transfected with Rfz1, M50 and either wild-type Dvl2 or a specific Dvl2 mutants. On the following day, assay for Lef/Tcf-sensitive transcription was performed. Statistical significance is provided, * denotes *p *< 0.005 (versus wild-type Dvl2 expressed cells). Results shown are mean values ± s.e.m. from 6 independent experiments.

Dvls have been reported to be substrates for phosphorylation by CK1δ/ϵ [15,16,21]. Phosphorylation is well-known to provoke protein-protein interactions; indirect evidence supports this same notion operating in Wnt signaling. For example, treating cells with serine/threonine phosphatase inhibitors (e.g., okadaic acid to selectively inhibit PP1 and PP2A) mimics Wnt action, affecting protein stability, activity, localization and trafficking of key elements including Dvls [24]. We sought direct evidence for the linkage between Dvl3 phosphorylation and Wnt action, interrogating the role of CK1δ in Wnt/β-catenin signaling. CK1δ was knocked-down in F9 cells. The cells, stably expressing rat Fz1 (Rfz1) and the M50 Super8xTOPFlash Lef/Tcf-sensitive transcriptional reporter, were treated with siRNA targeting expression of CK1δ. The CK1δ-deficient cells then were stimulated with Wnt3a. The read-out, Lef/Tcf-sensitive transcriptional activation, was assayed at 7 hr post Wnt stimulation. Knockdown of CK1δ severely attenuated Lef/Tcf-sensitive transcriptional activation in response to Wnt3a (Figure 1A). Treating cells with the IC261 compound, a selective inhibitor of CK1δ/ϵ, also severely blunted Lef/Tcf-sensitive transcription in response to Wnt3a (Figure 1B). Expression of a kinase-inactive mutant of CK1δ (K38M-CK1δ), like CK1δ depletion or chemical inhibition, blocked Lef/Tcf-sensitive transcription in response to Wnt3a. Basal transcriptional activity was similarly attenuated (Figure 1C). Conversely, expression of wild-type CK1δ dramatically potentiated Lef/Tcf-sensitive transcription. Expression of wild-type CK1δ rescued Wnt signaling in CK1δ-deficient cells (Figure 1D). These observations argue that CK1δ plays an essential role in the Wnt-responsive canonical pathway.

**Figure 2:**
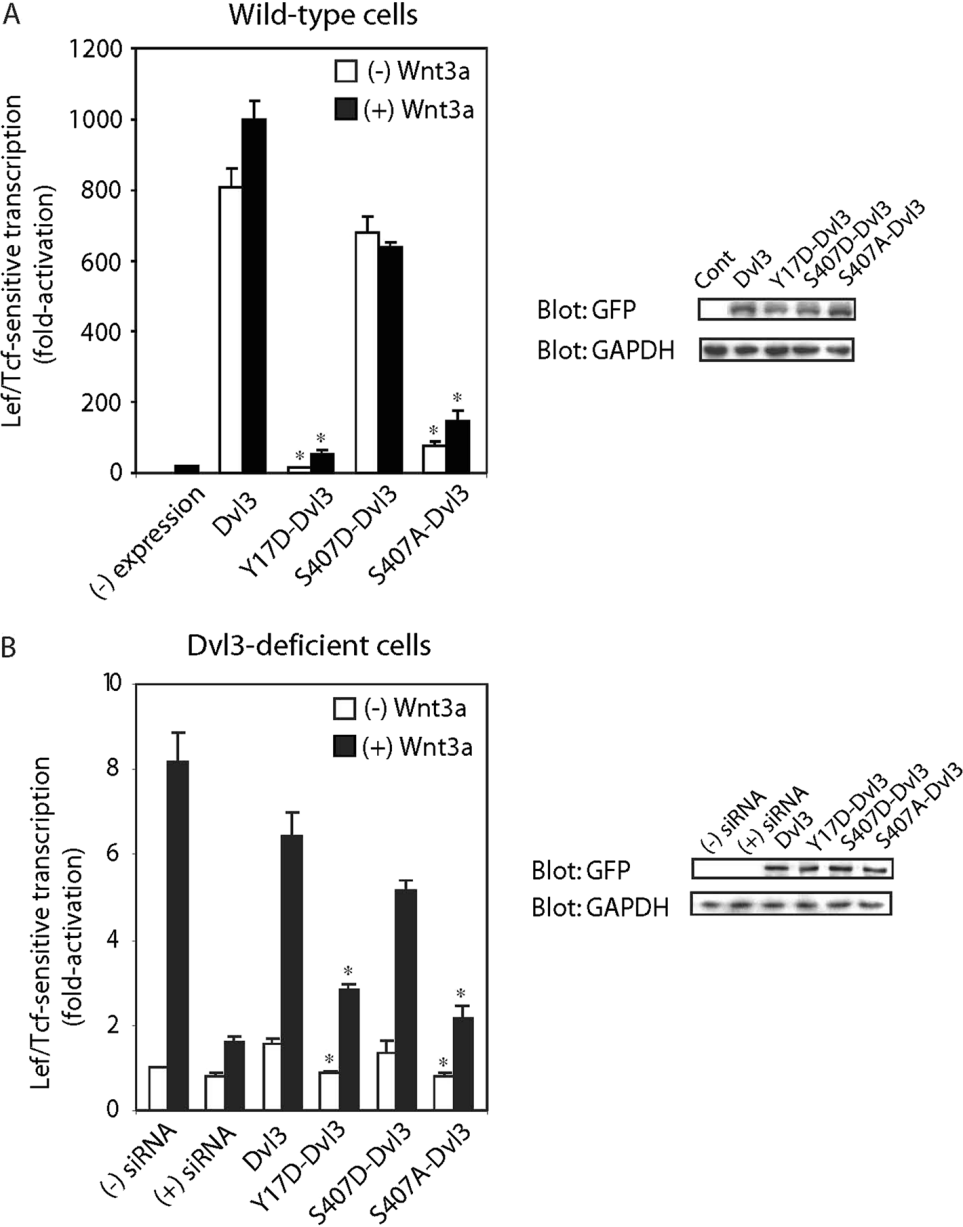
**Interrogation of the functional status of Y17 and S407 sites of Dvl3 in Lef/Tcf-sensitive transcriptional activation in response to stimulation with Wnt3a. ***Panel A*, F9 cells co-expressed with Rfz1, Super8xTOPFlash (M50) and either wild-type Dvl3 or one of following Dvl3 mutants (Y17D-Dvl3, S407A-Dvl3 and S407D-Dvl3) were stimulated with or without Wnt3a for 7 hr. Cell lysates were assayed for Lef/Tcf-sensitive transcription. Cell lysates were immunoblotted and subsequently stained with anti-GFP (Dvl3 expression) and anti-GAPDH (control) antibody. Results are displayed for transcriptional activity relative to unstimulated cells (set to “1”). Statistical significance is indicated (*, *p *< 0.005, versus Dvl3 expressed cells). *Panel B*, F9 cells stably expressing Rfz1 and M50 were treated with siRNA targeting Dvl3 one day before transfection of the cells with either wild-type Dvl3 or Y17D-Dvl3 or S407A-Dvl3 or S407D-Dvl3. On the following day, cells were stimulated with Wnt3a for 7 hr. Cell lysates were assayed for Lef/Tcf-sensitive transcription. Cell lysates were immunoblotted with either anti-GFP or anti-GAPDH antibody (control). Results are displayed for transcriptional activity relative to unstimulated cells (set to “1”). Statistical significance is indicated (*, *p *< 0.05, versus Dvl3 expressed cells).

Is there a direct linkage between Dvl3-mediated action and its phosphorylation? Eleven canonical sites are predicted as possible substrates for CK1δ-catalyzed phosphorylation in mouse Dvl3 (Additional file 1A). The first site of interest is S407. Scanning mutagenesis of Dvl2 was performed more readily and it revealed that S418 was as a site of CK1δ-catalyzed phosphorylation in this Dvl isoform (see Materials and Methods, Figure 1E). On this lead, the conserved serine, S407 in Dvl3, was selected for study. Alignment studies of Drosophila Dsh and the three mouse Dvl isoforms identified the second site of keen interest, Y17 (Additional file 1B). In support of this selection, the conserved Y27 of Dvl2 has been shown already to be necessary for normal Dvl polymerization/function [4]. This tyrosine residue in the DIX domain is conserved among all Dvl isoforms. Absent a known polymerization-defective mutant of Dvl3 to employ, we thus targeted Y17 in Dvl3. By focusing on these two sites (Y17 in regard to polymerization, and S407 in regard to phosphorylation), we sought to test the possible linkage between the phosphorylation and the assembly of Dvl3-based supermolecular complexes (Additional file 1B). The Y17D-Dvl3 mutant (analogous to Y27D-Dvl2, [4]) and the S407A-Dvl3 mutant were constructed and expressed. We studied the effects of loss of each site on function as well as the assembly of the Dvl3-based complexes (Figure 2). Immunoblotting of the whole-cell lysates with anti-green fluorescent protein (GFP) antibody established equivalence of expression of each construct. Staining with anti-glyceraldehyde-3-phosphate dehydrogenase (GAPDH, control) antibody established loading equivalence (Figure 2A, right panel). Lef/Tcf-sensitive transcription thus was normalized, based on Dvl expression as established by quantitative immunoblotting (Figure 2A, right panel).

Overexpression of wild-type Dvl3 alone can stimulate Lef/Tcf-sensitive transcription (Figure 2A). Expression of the alanine-substituted S407 Dvl3 (S407A-Dvl3), in contrast, failed to stimulate Lef/Tcf-sensitive transcription. Expression of the Y17D-Dvl3 mutant, like S407A, also failed to stimulate transcriptional activation. Aspartate-substituted S407D-Dvl3, harbors a new negative charge often phospho-mimetic with respect to phosphorylation of serine. S407D-Dvl3 expression stimulated Lef/Tcf-sensitive transcription as does overexpression of the wild-type Dvl3. Cells expressing either Dvl3 or S407D-Dvl3 mutant displayed no transcriptional activation in response to Wnt3a. Cells made deficient in Dvl3 *via* siRNA treatment fail to respond to Wnt3a, as expected (Figure 2B). Expression of wild-type Dvl3 fully rescued Lef/Tcf-sensitive transcription in the Dvl3-deficient cells. Expression of Y17D-Dvl3, in contrast, could not fully rescue the response. Expression of phosphorylation-mimetic mutant of Dvl3 (S407D-Dvl3) did effectively rescue Lef/Tcf-sensitive transcription. Expression of phosphorylation-defective mutant of Dvl3 (S407A-Dvl3) was unable to rescue the Lef/Tcf-sensitive transcription in response to Wnt3a (Figure 2B).

### Assembly of Dvl3-based supermolecular complexes: Effects of mutations

**Figure 3:**
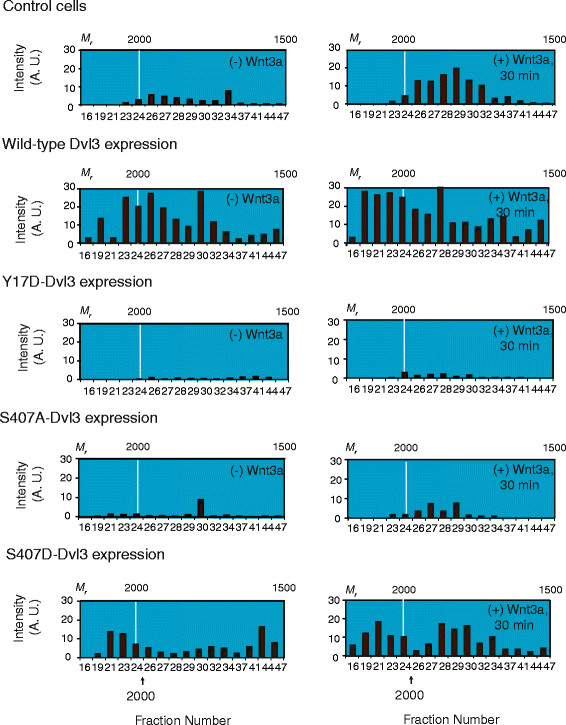
**Interrogation of functional roles of Y17 and S407 sites of Dvl3 in assembly of very large Dvl3-based supermolecular complexes in response to stimulation with Wnt3a. ***Y17D-Dvl3 and S407A-Dvl3 abolished the assembly of Dvl3-based supermolecular complexes in response to Wnt3, whereas S407D-Dvl3 enhances the assembly of Dvl3-based supermolecular complexes. *Cells expressing either wild-type Dvl3 or Y17D-Dvl3 or S407A-Dvl3 or S407D-Dvl3 were stimulated with or without Wnt3a for 30 min. Cells were lysed and extracts (20 mg protein) were applied to Sephacryl S-400 gel filtration column (AKTA, GE Health Care). Fractions were analyzed by SDS-PAGE and immunoblotted, subsequently stained with anti-Dvl3 antibody. Dvl3 blots were quantified by the calibrated scanner and results were displayed*. *The Dvl3-based supermolecular complexes in F9 cells expressing Rfz1 were also displayed as a control. The calculated, relative molecular weight (*M*_*r*_) positions from the calibration curve are labeled at the top. The bottom numbers indicate fraction number. Arrow indicates the precise position at which calibration proteins elute from Sephacryl S-400. Results are representative of at least 2 independent experiments.

Depletion of cellular Dvl3 as well as expression of Dvl3 S407A and Y17D mutants disrupt the signaling downstream of Frizzled to the level of Lef/Tcf- sensitive transcription. We sought to ascertain whether the basis for these loss-of-function mutations was proximal to the assembly of the very large, Dvl3-based supermolecular complexes [10]. Dvl3-based complexes can be molecularly sieved by size-exclusion chromatography (SEC) on large-pore matrices. F9 cells expressing wild-type Dvl3 or Dvl3 mutants were either unstimulated (−Wnt3a) or stimulated with Wnt3a (+Wnt3a) for 30 min and then harvested for subsequent SEC analysis. For analysis of Dvl3-based supermolecular complexes (~2 MDa-*M*_*r*_), whole-cell extracts were subjected to steric-exclusion chromatography on large-pore Sephacryl S-400 column. Sephacryl S-400 column chromatography resolves complexes with *M*_*r*_ ~ up to 10 MDa. High-resolution analysis complexes resolved on the lower *M*_*r*_ range of the chromatogram was performed on Superdex 200 matrix, targeting complexes of 75–800 kDa-*M*_*r*_. Fractions of each separation were analyzed by sodium dodecyl sulfate-polyacrylamide gel electrophoresis (SDS-PAGE) and quantitative staining of immunoblots probed with Dvl3-specific antibody (Additional file 2). SEC chromatograms were prepared from the lysates of cells without and with prior stimulation by Wnt3a (Figure 3, Additional files 2 and 3).

In the absence of Wnt3a, Dvl3-based complexes migrated primarily as one peak with ~2 MDa-*M*_*r*_. A second broader peak ranging from 100 ~ 500 kDa-*M*_*r*_ also was observed. Wnt3a treatment provoked a shift of the ~2 MDa-*M*_*r*_ complexes to much larger *M*_*r*_. The shift to higher *M*_*r*_ complexes in response to Wnt3a progressed for 60 min. The appearance of very large complexes was accompanied by a depletion of Dvl3-based complexes in the lower *M*_*r*_ 200–500 kDa range [10]. Temporally, Wnt3a-provoked assembly of Dvl3-based supermolecular complexes precedes Lef/Tcf-sensitive transcription activation (Figure 3). Expression of wild-type Dvl3 alone provoked an increase in the formation of very large (>2.0 MDa-*M*_*r*_) Dvl3-based complexes (Figure 3, second panel) as well as a subsequent activation of Lef/Tcf-sensitive transcription in the absence of Wnt3a (Figure 2A). In contrast, expression of Y17D-Dvl3 abolished both the formation of very large Dvl3-based supermolecular complexes and the activation of Lef/Tcf-sensitive transcription (Figure 3, third panel and Figure 2A). This blockade, induced by the expression of the Y17D-Dvl3 mutant, was obvious in the absence as well as the presence of Wnt3a. The majority of the Dvl3-based complexes formed in cells expressing Y17D-Dvl3 displayed *M*_*r*_ of only 100 ~ 200 kDa (Additional file 3, third panel).

For cells expressing S407A-Dvl3, Wnt3a was unable to provoke assembly of Dvl3-based supermolecular complexes [(~2.0 MDa-*M*_*r*_), Figure 3, fourth panel and Additional file 2)]. The failure to assemble very large Dvl3-based complexes in S407A Dvl3-expressing cells yields a molecular explanation for the inability of the cells to activate of Lef/Tcf-sensitive transcription (Figure 3, fourth panel, Additional file 2 and Figure 2A). Expression of S407D-Dvl3, like wild-type Dvl3, provoked assembly of Dvl3-based supermolecular complexes. Upon Wnt3a stimulation, a modest upfield shift of Dvl3-based supermolecular complex *M*_*r*_ was observed (Figure 3, fifth panel and Additional file 2). Importantly, it was observed that the expression of each of Dvl3 mutants did not affect the assembly of either Dvl1-based or Dvl2-based supermolecular complexes (results not shown). Expression of S418A-Dvl2 mutant (the Dvl2 analog of S407A-Dvl3) also was probed. Expressing S418A-Dvl2 was found to attenuate the assembly of Dvl3-based supermolecular complexes. Only the smaller 100 ~ 300 kDa-*M*_*r*_ complexes accumulated, but not the very large >2 MDa-*M*_*r*_ Dvl3-based complexes (Additional file 4A and 4B). These data agree well with earlier studies on the complex interplaying of Dvl isoforms in signaling from Fz to Lef/Tcf-sensitive transcription [14].

**Figure 4:**
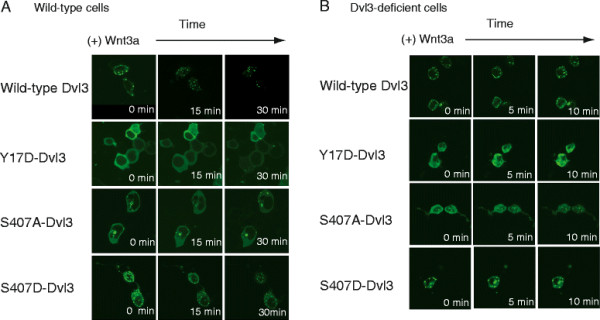
**Interrogation of functional roles of Y17 and S407 sites of Dvl3 in formation of macromolecular Dvl3-based punctae in response to stimulation with Wnt3a. ***Panel A, live cell images of expression of either wild-type Dvl3 or Y17D-Dvl3 or S407A-Dvl3 or S407D-Dvl3 in response to Wnt3a. *F9 cells were co-transfected with Rfz1 and either wild-type Dvl3 or Y17D-Dvl3 or S407A-Dvl3 or S407D-Dvl3. One day post transfection, cells were stimulated with Wnt3a and cells images were taken at 0, 15, and 30 min post stimulation with Wnt3a. *Panel B, live cell images of cells expressing either wild-type or Dvl3 mutants (Y17D-Dvl3 or S407D-Dvl3 or S407D-Dvl3) in Dvl3-deficient cell: response to Wnt3a. *F9 cells stably expressing with M50 and Rfz1 were treated with siRNA targeting Dvl3. On the following day, cells were transfected with either wild-type Dvl3 or one of the Dvl3 mutants. Post 24 hr of transfection, cells were stimulated with Wnt3a and live cell images were captured during Wnt stimulation. Results were displayed at 0, 5, and 10 min time points. The images shown are representative of three or more separate experiments.

Do punctae observed widely by fluorescence microscopy of cells expressing GFP-tagged Dvls constitute these same dynamically assembled, Dvl-based supermolecular complexes? We tested this possibility directly. F9 cells expressing GFP- and HA-dually tagged wild-type Dvl3 were subjected to fluorescence microscopy. Cells were either untreated or treated with Wnt3a over the course of 30 min. Image of live cells that express wild-type and mutant Dvl3 were observed at 0, 15, and 30 min (Figure 4A). For GFP-tagged wild-type Dvl3, punctate cellular structures were observed and localized at the cell membrane. Wnt3a stimulated translocation of the punctae away from the cell membrane. For cells expressing Y17D-Dvl3, only diffuse cytoplasmic distribution of GFP-tagged Dvl3 was observed. Enrichment of large, membrane-associated punctae in response to Wnt3a was not observed. These fluorescence images would be consistent with polymerization-defective Dvl3, observed in either the absence or the presence of Wnt3a. Cell expressing of S407D-Dvl3, the phospho-mimetic mutant, displayed punctate structures in the absence of Wnt3a similar to those formed in cells expressing wild-type Dvl3 following Wnt3a stimulation. Punctae are found at the cell membrane as well as in the cytoplasm. Wnt3a treatment failed to significantly alter the distribution of the punctae of S407D-Dvl3 expressing cells. In comparison to wild-type Dvl3, S407A-Dvl3 mutant displayed a sharp reduction in both number and size of the punctae. Significant amounts of S407A-Dvl3 mutant remained diffused throughout the cytoplasm. Wnt3a treatment did not induce any change of the apparent size and location of such small punctae composed of the S407A-Dvl3 mutant.

To address the issue of overexpression, we performed parallel experiments in Dvl3-deficient cells (siRNA treatment, Figure 4B). Punctae formation in cells expressing wild-type Dvl3 or S407D-Dvl3 was obvious. The distribution of punctae changed during Wnt3a stimulation. For cells expressing the Y17D-Dvl3, a diffuse distribution of GFP-tagged Dvl3 was observed. Wnt3a failed to change the cellular distribution of Y17D-Dvl3. Cells expressing S407A-Dvl3 displayed few punctae, diffused in distribution. We probed the same question in a different cellular context. Parallel experiments were performed in human embryonic kidney cells (HEK293). Images from fluorescence microscopy of GFP-tagged wild-type and Dvl3 mutants expressed in HEK293 displayed the same character as those observed in F9 cells (Additional file 5).

**Figure 5:**
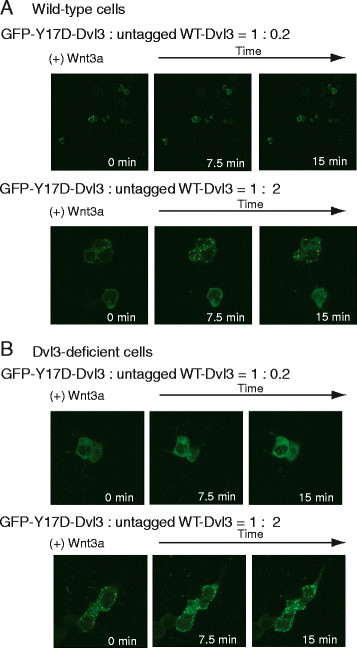
**Formation of Dvl3-based punctae by wild-type versus mutant forms of Dvl3. ***Y17D-Dvl3 forms punctae in cells co-expressing wild-type Dvl3 in untreated and Dvl3-deficient F9 cells*. *Panel A*, F9 cells were co-transfected with GFP- and HA-tagged Y17D-Dvl3 and increasing amount of co-expressed untagged wild-type Dvl3 (ratio = 1 : 0.2, or 1 : 2). Post 1 day transfection, cells were stimulated with Wnt3a. Cell images were taken during Wnt stimulation. Results were displayed at 0, 7.5, and 15 min time-points. *Panel B*, parallel experiments were performed in F9 cells made deficient of endogenous wild-type Dvl3 by siRNA. The images shown are representative of three or more separate experiments.

We interrogated whether the Dvl3 punctae of cells expressing Dvl3 mutants were affected by expressing increasing amount of wild-type Dvl3. Cells expressing a constant level of GFP-tagged Y17D-Dvl3 were co-transfected so as to express increasing amounts of wild-type Dvl3 also (Figure 5A and 5B). Transfection of GFP-tagged Y17D-Dvl3 alone displayed a diffuse cytoplasmic distribution and no punctae. Co-transfection of wild-type Dvl3 (untagged) with the GFP-tagged Y17D-Dvl3 provoked formation of the punctae. We compared punctae formation in wild-type and Dvl3-deficient cells. Formation of the punctae was found to be dependent on the amount of wild-type Dvl3 co-expressed with the Y17D-Dvl3 (i. e., in the presence of endogenous Dvl3 vs. Dvl3-deficient, Figure 5A and 5B). In Dvl3-deficient cells, only cells expressing a high ratio (i. e., 1:2) of wild-type Dvl3 toY17D-Dvl3 displayed punctae (Figure 5B). Wnt3a provoked changes in the macroscopic size and cellular localization of punctae (Figure 5A and 5B). Similar results were obtained when cells were co-transfected in a manner to express a low, constant amount of GFP-tagged S407A-Dvl3 mutant and increasing amounts of wild-type Dvl3 (results not shown). Thus wild-type Dvl3 clearly is capable of forming complexes with Dvl3 harboring the Y17 and S407 mutations.

### CK1 modulates the formation of punctae

**Figure 6:**
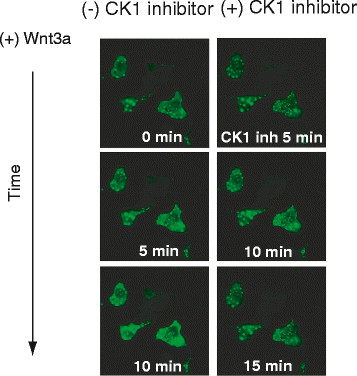
**CK1δ/ϵ regulates formation of Dvl3-based very large punctae. ***Treatment with CK1δ/ϵ inhibitor decreases number as well as apparent size of the punctae. *F9 cells were co-transfected with Rfz1 and GFP- and HA-tagged wild-type Dvl3. Cells were stimulated with Wnt3a and images of live cells were captured at the times indicated. After 10 min post Wnt stimulation, a CK1δ/ϵ selective inhibitor (IC261, 5 μM) was added and cell images captured continuously. Results displayed are images captured at 5, 10, and 15 min post treatment with IC261. The images displayed are representative of two or more separate experiments.

Expression of the phospho-mimetic mutant of Dvl3 (S407D-Dvl3) provokes assembly of Dvl3-based supermolecular complexes, punctae formation and Lef/Tcf-sensitive transcription. Consequently, we tested if inhibition of CK1δ/ϵ activities affects formation of Dvl3-based punctae. GFP-tagged wild-type Dvl3 was expressed in cells and the cells treated with Wnt3a. A CK1δ/ϵ−selective inhibitor (IC261) was added to the cell cultures at 10 min post Wnt stimulation. Live cell images were taken continuously (Figure 6). As expected, Wnt3a stimulated formation of larger size punctae, in a time dependent manner. Of interest, treatment cells with IC261 provoked a reversal of punctae formation. IC261 treatment yields a reduction in distribution and apparent size of Dvl3 punctae in response to Wnt3a (Figure 6).

### Axin is essential for the assembly of Dvl3-based supermolecular complexes

**Figure 7:**
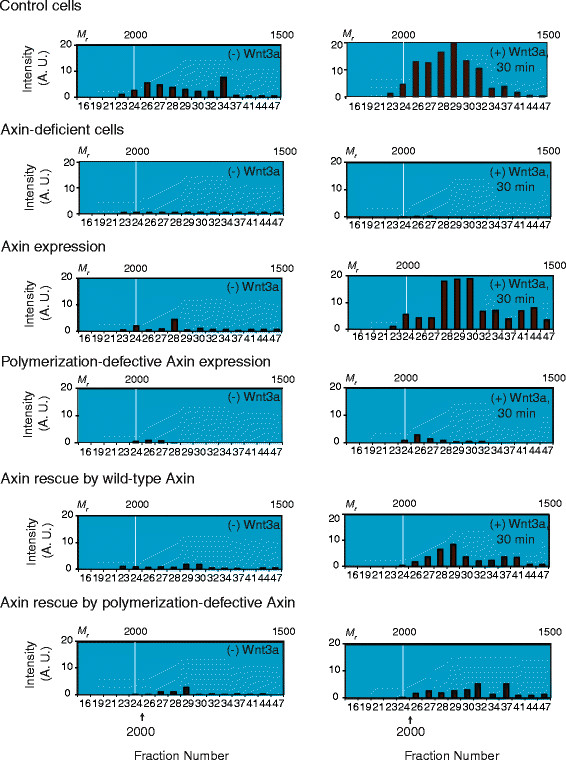
**Expression of Axin M3 mutant precludes assembly of very large Dvl3-based supermolecular complexes in response to Wnt3a. Control cells**, *Wnt3a stimulates formation of very large M*_*r*_*-complexes*. SEC chromatogram of Dvl3-based complexes prepared from untreated cells. **Axin-deficient cells, ***knockdown of Axin attenuates assembly of Dvl3-based supermolecular complexes in response to Wnt3a. *F9 cells were transfected with siRNA targeting Axin one day before transfection with Rfz1. Twenty four hr later, cells were either unstimulated or stimulated with Wnt3a for 30 min. **Axin expression**, *expression of *w*ild-type Axin alone stimulates assembly of Dvl3-based supermolecular complexes. *F9 cells were co-transfected with Rfz1 and expression vectors harboring human wild-type Axin. Two days post transfection, F9 cells were treated either without or with Wnt3a for 30 min. **Polymerization-defective Axin expression**, *expression of M3 Axin mutant blocks assembly of very large Dvl3-based supermolecular complexes in response to Wnt3a. *F9 cells were co-transfected with Rfz1 and expression vectors harboring human M3 Axin mutant. Two days post transfection, F9 cells were treated either without or with Wnt3a for 30 min. **Rescue of Axin-depletion by expression of wild-type Axin**, *expression of *w*ild-type Axin rescues the inability of Axin-deficient cells to assemble Dvl3-based supermolecular complexes in response to Wnt3a. *Cells were treated with siRNA targeting Axin one day before subsequent co-transfection with Rfz1 and human wild-type Axin for an additional day. Twenty four hr after this final transfection, cells were either unstimulated or stimulated with Wnt3a for 30 min. **Axin rescue by polymerization-defective Axin**, *expression of M3 Axin fails to rescue the inability of Axin-deficient cells to assembly of Dvl3-based supermolecular complexes in response to Wnt3a. *Cells were treated with siRNA targeting Axin one day before subsequent co-transfection with Rfz1 and M3 Axin mutant for an additional day. Twenty four hr after this final transfection, cells were either unstimulated or stimulated with Wnt3a for 30 min. For all experiments, cell lysates were subjected to Sephacryl S-400 gel filtration column chromatography (SEC) and resolved fractions were analyzed by SDS-PAGE, immunoblotting and eventual staining with anti-Dvl3 antibody. Dvl3 blots were subjected to quantification by calibrated scanning and the results displayed are representative of at least 2 independent experiments, which yielded quantitatively similar results. The calculated, relative molecular weight (*M*_*r*_) positions from the calibration curve are labeled at the top of each SEC chromatogram. The bottom labels indicate fraction number. Arrows indicate the precise position at which calibration protein elute from Sephacryl S-400.

Inhibition of the Axin destruction complexes by Wnt3a requires both Axin and Dvls [7,28,30]. We interrogated whether assembly of very large Dvl3-based supermolecular complexes in response to Wnt is dependent on Axin. As noted by others [35], knockdown of Axin stimulates Lef/Tcf-sensitive transcriptional activation (result not shown). In control cells, Wnt3a stimulation provoked assembly of Dvl3-based supermolecular complexes (~2 MDa-*M*_*r*_, Figure 7). In Axin-deficient cells, Dvl3-based complexes were smaller, migrating as two broad peaks centered around 1 and 0.6 MDa-*M*_*r*_. In the absence of Axin, Wnt3a failed to provoke the assembly of large Dvl3-based supermolecular complexes (~2 MDa-*M*_*r*_) (Figure 7 and Additional files 6 and 7).

### Axin regulates the assembly of very large Dvl3-based supermolecular complexes

Since Axin and Dvls interaction is critical for Wnt/β-catenin signaling, we further interrogated the influence of Axin on the assembly of the very large Dvl3-based supermolecular complexes. Our goal was to focus attention of the formation of Dvl3-based supermolecular complexes that occurs only in response to Wnt3a. Overexpression of Axin provokes the formation of Dvl-based punctae [5]. Axin mutant M3, carries two point mutations (I758A, R761D) of residues in the N-terminal reach of the conserved DIX domain. Axin M3 expression has been shown to block the self association of Axin. The M3 Axin is unable to polymerize with Dvl and unable to support Wnt signaling [36]. We first examined cells expressing low levels of either Axin (i. e., less than 7% of the cellular complement of Axin) or similar levels of the M3 Axin mutant. Such modest expression of wild-type Axin itself provoked the assembly of Dvl3-based supermolecular complexes in Wnt3a-stimulated cells (Figure 7 and Additional files 6 and 7). Expression of M3 Axin, in sharp contrast, reduced the abundance of Dvl3-based complexes over the entire range of *M*_*r*_ in the SEC chromatograms (Additional file 7). More importantly, cells expressing M3 Axin failed to form the very large Dvl3-based supermolecular complexes in response to Wnt3a (Figure 7 and Additional file 6).

### In axin-deficient cells, axin, but not M3 axin, rescues the assembly of Dvl3-based complexes

We next compared the ability of wild-type Axin versus M3 Axin to rescue the loss of formation of very large Dvl3-based complexes observed in cells made deficient of Axin by siRNA treatment (Figure 7 and Additional files 6 and 7). Expression of wild-type Axin in Axin-deficient cells fully restored the assembly of the two dominant peaks of Dvl3 complexes (i.e., 0.2-0.5 and ~2 MDa-*M*_*r*_) in untreated cells (Additional file 7). Expression of wild-type Axin also rescued the ability of Wnt3a to provoke assembly of the very large Dvl3-based supermolecular complexes (Figure 7 and Additional file 6). The polymerization-defective M3 Axin mutant, however, failed to significantly rescue the loss of Dvl3-based supermolecular complexes with ~2 MDa-*M*_*r*_. Few large complexes were detected in cells expressing M3 Axin (Figure 7 and Additional file 6). This M3 Axin-dependent failure to rescue assembly of Dvl3-based supermolecular complexes was not overcome by stimulation with Wnt3a (Figure 7). Only Axin, but not M3 Axin, readily rescued Axin-deficient cells with respect to both assembly of very large Dvl3-based complexes (shown) and Wnt3a activation of Lef/Tcf-sensitive transcription (not shown).

## Discussion

We report that protein phosphorylation of Dvl3 by CK1δ is essential to Wnt3a action and to the proper assembly of very large *M*_*r*_, Dvl3-based supermolecular complexes. Dvls interact with CK1ϵ *via* PDZ/DEP domains [17,18,20]. Phosphorylation of Dvl3 S407, for example, is shown to be obligate for the assembly of the very large Dvl3-based complexes, punctae formation, and Wnt/β-catenin signaling itself. Phosphorylation-mimetic mutant of Dvl3 (S407D-Dvl3) provoked prominent assembly of very large Dvl3-based supermolecular complexes, formation of Dvl3-based punctae, as well as activation of Lef/Tcf-sensitive transcription, in the absence of Wnt3. Similarly a phosphorylation-defective mutant of Dvl3 (S407A-Dvl3) was unable to support assembly of very large Dvl3-based supermolecular complexes, Dvl3-based punctae formation, and Lef/Tcf-sensitive transcription in response to Wnt3a. S407 is located between SH3-binding domain and DEP domain, in close proximity to CK1δ binding sites. Our studies demonstrate that phosphorylation of S407 is essential to supermolecular formation and Wnt/β-catenin signaling. A recent report that phosphorylation of Dvl by CK1δ/ϵ stimulates the oligomerization of Dvl [37] is confirmed and extended herein. Hyper-phosphorylated Dvl (i.e., phosphorylated and gel-shifted Dvl) has less ability to self-associate [37]. The observation that hyper-phosphorylated Dvl accumulates within 2 hr of Wnt3a stimulation agrees well with our results on the assembly of very large (~2 MDa-*M*_*r*_) Dvl3-based supermolecular complexes. The assembly of the Dvl3-based supermolecular complexes is a comparatively early response to Wnt stimulation [10].

Does polymerization of Dvl and punctae formation (detected by fluorescence microscopy) reflect the assembly of Dvl3-based supermolecular complexes? We show conclusively that formation of punctae correlates well with the formation of Dvl3-based supermolecular complexes. Expression of mutants of Dvl that attenuate Wnt3a-induced assembly of very large (~2 MDa-*M*_*r*_) supermolecular complexes blocks both the ability of Wnt3a to stimulate Dvl-based punctae and Lef/Tcf-sensitive transcriptional activation. Schwarz-Romond et al. first reported that a “polymerization-defective mutant of Dvl2”, Y27D-Dvl2, displayed no large punctae, rather only a diffuse, cytoplasmic distribution of Dvl2 [4]. Furthermore expression of Y27D-Dvl2 led to an inability of the cells to show Lef/Tcf-sensitive transcription in response to Wnt3a [4]. Expression of the analogous mutation in Dvl3, Y17D-Dvl3, likewise displayed no punctae formation, no assembly of Dvl3-based supermolecular complexes, and no ability to stimulate Lef/Tcf-sensitive transcriptional activation in response to Wnt3a.

In the case of Dvl2, the importance of tyrosine residue (Y27) has been mapped to the β2 and β4 interaction [4]. Mutation of Y27 in β2 surface effectively blocked the β2-β4 interaction [4]. The Y27 residue in the DIX domain of Dvls is thought to play an important role in enabling the formation of Dvl-based punctae. We show that loss-of-function mutations of the analogous site in Dvl3 (Y17) preclude the assembly of very large Dvl3-based supermolecular complexes in response to Wnt3a. These Dvl3 mutants do not lose their ability to from complexes with wild-type Dvl3. Head-to-tail association for Dvls has been proposed in a previous report [4]. Similarly, inhibition of CK1δ/ϵ provoked a sharp reduction in apparent size and distribution of the punctae and blocked Lef/Tcf-sensitive transcription.

Axin organizes APC, GSK3β, CK1α and β-catenin to assemble large multiprotein complexes essential to the destruction of the β-catenin. The DIX domain at C-terminus of Axin mediates both Axin homo-dimerization [25-27] as well as heterodimerization of Axin with Dsh/Dvl [7,28,29]. Dvl mediates Wnt3a signaling, interacting with Axin to block the ability of β-catenin to be phosphorylated and degraded [7,28,30,38]. By fluorescence microscopy, Axin has been localized to large punctae [5]. The abundance of Axin is kept quite low. Axin may act catalytically in Wnt signaling to activate Lef/Tcf-sensitive transcription [34]. Knockdown of Axin attenuated assembly of Dvl3-based supermolecular complexes in response to Wnt3a and also blocked assembly of very large punctae. Wild-type Axin is readily rescues the assembly of Dvl3-based supermolecular complexes in Axin-deficient cells. Expression of the polymerization-defective M3 Axin mutant, in contrast, failed to rescue the ability of Wnt3a to assemble the Dvl3-based supermolecular complexes. Our studies, like earlier studies [4,36], show that mutations of DIX domain of either Axin or of Dvl provoke the formation of diffuse, relatively small cytoplasmic punctae. Expression of wild-type Axin, in contrast, supports formation of very large punctae as well as complexes characterized as very large by SEC.

Assembly of Dvl3-based supermolecular complexes is an early response to Wnt, preceding Wnt stimulation of Lef/Tcf-sensitive transcription. Proteomic analyses of Dvl3-based supermolecular complexes separated by SEC identify CK1α, CK1γ, CK2α and β, LRP6, PI4 kinase and Src [39]. The proteomic data derived from isolated supermolecular complexes reinforces and extends the functional significance of Dvl3-based supermolecular complexes [39]. The upper limit of Dvl3-based supermolecular complexes has been amended recently to *M*_*r*_ = 35 MDa, based upon estimates recalibrated from fluorescence correlation microscopy in live cells [40]. Formation of very large Dvl3-based supermolecular complexes is an early step in supermolecular complexes and punctae formation (which are likely composed of multimers of the very large complexes) essential to Wnt/β-catenin signaling.

## Materials and methods

### Materials

The following reagents were purchased from the indicated commercial supplier(s): anti-Dvl1 and anti-Dvl2 antibodies from Santa Cruz Biotechnology (Santa Cruz, CA); anti-GSK3β and anti-Dvl3 antibodies from Cell Signaling (Danvers, MA); Immobilon membrane from Millipore (Bedford, MA); purified, biologically active Wnt3a, and anti-Axin antibody from R& D Systems (Minneapolis, MN). The plasmid for human GFP- and HA-tagged M3-Axin (polymerization defective Axin) was kind gifts from Dr. Mariann Bienz (MRC Laboratory of Molecular Biology, Cambridge Cancer Center, University of Cambridge, UK). The plasmids for CK1δ and K38M-CK1δ were kind gifts from Dr. D. W. Meek (Biomedical Research Institute, Ninewells Hospital, University of Dundee, Dundee, Scotland, UK).

### Cell culture and transfection

Mouse F9 teratocarcinoma cells (F9) cells, transfected with a pcDNA3 expression vector harboring rat Frizzled-1 (Rfz1), were grown in Dulbecco’s modified Eagle’s medium (DMEM) as described previously [24]. Cells were transiently transfected using lipofectamin (Invitrogen, Carlsbad, CA). For overexpression studies, GFP- and hemagglutinin (HA)-tagged mouse Dvl3 or mutants of GFP- and HA-tagged mouse Dvl3, wild-type or mutant Axin were transiently transfected into F9 cells with Rfz1 48 hr before Wnt3a stimulation.

### Identification of phosphorylation sites for CK1δ

Histidine tagged Dvl2 was expressed in Sf9 cell and purified by Ni^2+^-affinity chromatography [41]. Phosphorylation of rDvl2 by CK1δ was carried out in a kinase buffer for 1 hr. Phosphorylated rDVl2 were digested with trypsin and analyzed by liquid chromatography electrospray ionization mass spectrometry (LC-ESI-MS-MS). 15 phosphorylation sites (T25, T94, S106, S141, S155, S158, S281, S286, S298, S329, S358, S418, S425, S651 and S709) were identified. To evaluate potential sites of phosphorylation, we mutated Dvl2 (serine/threonine to alanine mutation) using Quickchange Mutagenesis System (Stratagene, La Jolla, CA). The ability of each mutant to affect Lef/Tcf-sensitive transcription was investigated (Figure 1E). Mutation of serine to alanine on S286 and S418 (S286A- and S418A-Dvl2) dramatically reduced Lef/Tcf-sensitive transcription. T25A-Dvl2 displayed only a modest inhibition of Lef/Tcf-sensitive transcription. Previously, two sites on Dvl1 phosphorylation by CK1ϵ, (S139 and S142), were identified [42]. Mutation of these sites did not abolish Dvl activity [42]. Inhibition of Lef/Tcf-sensitive transcription by mutations (serine to alanine) of S155 and S158 (conserved sites of Dvl1) of Dvl2 or double mutations of S155/158 was not observed (Figure 1E).

### Live-cell imaging of Dvl3 and Dvl3 mutants

F9 cells were plated on glass-bottom dishes (*In Vitro* Scientific, USA) pre-coated with laminin overnight at 37 °C in a CO_2_ incubator. HEK293 cells were plated on the same dishes, pre-coated with collagen for 1 hr at 37 °C in a dry incubator. The cells were co-transfected with Rfz1 and either wild-type or mutant of GFP- and HA-tagged mouse Dvl3. Twenty four hr later, the cells were stimulated with Wnt3a (20 ng/ml) and live images taken every minute in the course of 30 min using a confocal laser scanning microscope Olympus FluoView 1000 and commercial software. Where indicated, cells were treated with either 5 μM IC281 or vehicle consequent to Wnt stimulation.

### Knockdown of axin by siRNA

siRNA sequences designed to suppress Axin are as follows: siRNA sequences targeting Axin, AGGUCUAGCUGAAUUAUGGtt and CCAUAAUUCAGCUAGACCUtg; one-day after treatment with siRNA targeting Axin, cells were transfected with an expression vector harboring Rfz1. For some experiments, cells were co-transfected with Rfz1 and either human wild-type Axin or M3 Axin mutant. On the following day, F9 cells were stimulated with or without Wnt3a for the indicated time. For Lef/Tcf-sensitive transcription, F9 cells stably expressing Rfz1 and Super8xTOPFlash (M50) were transfected with siRNA targeting Axin one day before co-transfection with either human wild-type Axin or mutant of Axin. Cells thereafter were stimulated without and with Wnt3a. Read-outs of Dvl3-based supermolecular complex assembly (assayed by SEC), formation of Dvl3-based punctae (assayed by fluorescence microscopy), and Lef/Tcf-sensitive transcriptional activation (assayed using the M50 reporter for β-catenin) were measured at the appropriate times following Wnt3a treatment.

### Expression of axin

We employed Axin expression at 1.5 μg DNA of expression vector/150 mm dish, which does not induce on its own Lef/Tcf-sensitive transcriptional activation. The M3 Axin mutant (I758A, R761D) was employed in expression studies under the same conditions [35]. Cells were co-transfected with an expression vector harboring the Rfz1 and either wild-type Axin or M3 mutant of Axin. Post 48 hr of transfection, cells were stimulated or unstimulated with Wnt3a for 30 min. Cell lysates were subjected to SEC and resolved fractions of the chromatogram were analyzed by SDS-PAGE, immunoblotting, and stained with anti-Dvl3 or anti-Axin antibodies, as indicated.

### Assay for lef/tcf-sensitive transcriptional activation

F9 cells were grown on 12–well plates and co-transfected with Rfz1 and Super8xTOPFlash (M50) one day before stimulation cells with or without Wnt3a (20 ng/ml) for 7 hr. Cells were harvested and prepared using lysis buffer [12.5 mM Tris-H_3_PO_4_ pH 7.8, 1 mM Trans-1, 2-cyclohexanediaminetetraacetic acid (CDTA), 2 mM DTT, 10% glycerol and 1% Triton X-100 (Promega, Madison, WI)]. The luciferase assay was performed as previously described [13]. Results were displayed relative to those of the unstimulated cells (i.e., control cells were set to “1”). For the experiments with Dvl3 mutants, the expression levels of each mutant were normalized by quantifying the amount of the GFP-tag which was incorporated into each construct. These normalized results are displayed.

### Separation of dvls-based supermolecular complexes by steric-exclusion chromatography

Dvl3-based supermolecular complexes are separated by Sephacryl S-400 gel filtration column (HiPrep Sephacryl S-400 High resolution column, fast-performance AKTA liquid chromatography, GE Healthcare). When higher resolution was desired to analyze lower *M*_*r*_–species, Superdex 200 gel filtration column was employed (HiLoad Superdex ^TM^ 200 prep grade 26/60, fast-performance liquid chromatography system AKTA, GE Healthcare), as previously reported [10]. Briefly, F9 cells co-expressing Rfz1 and either wild-type Dvl3 or mutants of Dvl3, or wild-type Axin or Axin mutant were stimulated or unstimulated with Wnt3a for 30 min. Cells were harvested and suspended in ice-cold buffer (20 mM Tris–HCl pH 8.0, 0.2 M NaCl, 1% NP-40, 1 mM PMSF, 10 μg/ml leupeptin, and 10 μg/ml aprotinin) and disrupted by repeated passage through a 23-gauge needle, and then centrifuged. Supernatants were filtered, diluted with buffer containing no detergent and applied to either the Sephacryl S-400 or the Superdex 200 gel filtration column which was preequilibrated with 20 mM Tris–HCl (pH 8.0), 0.2 M NaCl, and 10% glycerol. Each fraction was analyzed by SDS-PAGE, immunoblotting, and stained with anti-Dvl3 body. Dvl3 blots were quantified and results displayed. The expression levels of each mutant were normalized based on the results of quantification of expression rendered by immunoblots stained with anti-GFP antibody. Protein concentration was determined by use of the Bradford assay.

### Immunoblotting

Proteins were analyzed by SDS-PAGE and immunoblotting [13]. Immune complexes were made visible using a horseradish peroxidase-conjugated, secondary antibody in tandem with ECL chemiluminescence.

### Quantification of stained bands on immunoblots

Immunoreactive bands were scanned by calibrated Umax 1000 scanner equipped with SilverFast software (LaserSoft Imaging Inc, Longboat key, FL). The bands were quantified by using Aida software (Raytest, Germany).

## Abbreviations

Fz, Frizzled; Dsh/Dvl, Dishevelled; Fcs, Fluorescence correlation microscopy; CK, Casein kinase; GSK3, Glycogen synthase kinase 3; APC, Adenomatous polyposis coli; SDS-PAGE, Sodium dodecyl sulfate-polyacrylamide gel electrophoresis; Rfz1, Rat Fz1; M50, Super8xTOPFlash; GFP, Green fluorescent protein; GAPDH, Glyceraldehyde-3-phosphate dehydrogenase; SEC, Size exclusion chromatography; F9 cells, Mouse F9 teratocarcinoma cells; HEK cells, Human embryonic kidney cells; DMEM, Dulbecco’s modified Eagle’s medium; HA, Hemagglutinin.

## Competing interests

The author(s) declare that they have no competing interests.

## Author’s contributions

NY designed the study, analyzed the data and drafted the manuscript. NY and NGM carried out the cell image analysis. HY and CCM provided support and coordination, editing various drafts of the manuscript. All authors read and approved the final manuscript.

## Supplementary Material

Additional file 1 **Predicted sites of phosphorylation catalyzed by CK1δ on Dvl3. ***Panel A, Putative phosphorylation sites by CK1δ. *Mouse Dvl3 domains are shown: DIX domain, yellow; PDZ domain, pink; DEP domain, blue. Putative phosphorylation sites are shown by bold character. Red characters display mutation employed in this study. *Panel B, Alignment of Dsh and mouse Dvls. *Sequences displayed are those flanking predicted phosphorylation sites of interest on Dvl3. Conservation of residues, corresponding to residues Y17 and S407 of Dvl3 are projected onto Dsh and Dvl isoforms, blocked written squares. Click here for file

Additional file 2 **The assembly of Dvl3-based supermolecular complexes in response to expression of Dvl3 mutants: immunoblot data. ***Expression of eitherY17D-Dvl3 or S407A-Dvl3 abolishes assembly of Dvl3-based supermolecular complexes, whereas expression of S407D-Dvl3 enhances assembly of Dvl3-based supermolecular complexes in response to Wnt3a. *Cells expressing either wild-type Dvl3 or Y17D-Dvl3 or S407A-Dvl3 or S407D-Dvl3 were stimulated either with or without Wnt3a for 30 min. Cells were lysed and extracts (20 mg protein) were applied to Sephacryl S-400 gel filtration column (AKTA, GE Health Care). Fractions were analyzed by SDS-PAGE and immunoblotted with anti-Dvl3 antibody. Blots are shown in the region with *M*_*r *_greater than1.5 MDa. Top labels specify the fraction number. Click here for file

Additional file 3 **Interrogation of Y17 and S407 sites of Dvl3 in the context assembly of lower- *M***_***r ***_**Dvl3-based supermolecular complexes in response to Wnt3a: SEC analysis on Superdex 200. ***Y17D-Dvl3 and S407A-Dvl3 abolishes the assembly of Dvl3-based supermolecular complexes in response to Wnt3, whereas expression of S407D-Dvl3 enhances the assembly of Dvl3-based supermolecular complexes. *Cells expressing either wild-type Dvl3 or Y17D-Dvl3 or S407A-Dvl3 or S407D-Dvl3 were stimulated either with or without Wnt3a for 30 min. Cells lysates (20 mg protein) were analyzed by SEC. The Dvl3-based complexes with *M*_*r*_ ≤ 1.5 MDa were characterized by Superdex 200 gel filtration column (AKTA, GE Health Care). Fractions were analyzed by SDS-PAGE and immunoblotted with anti-Dvl3 antibody. Dvl3 blots were quantified by the calibrated scanner and results were displayed*. *The Dvl3-based supermolecular complexes in F9 cells expressing Rfz1 were also displayed as a control. The calculated, relative molecular weight (*M*_*r*_) positions from the calibration curve are labeled at the top. The bottom numbers indicate fraction number. Arrows indicate the precise position at which calibration proteins elute from Superdex 200. Results are representative of at least 2 independent experiments. Click here for file

Additional file 4 **Expression of S418A-Dvl2 abolishes the assembly of Dvl3-based supermolecular complexes. **F9 cell were co-transfected with Rfz1 and either wild-type Dvl2 or S418A-Dvl2. Two days post transfection, cells were either treated or untreated with Wnt3a for 30 min. Cell lysates were subjected to characterization by SEC on Superdex 200 matrixes. Fractions separated by SEC were analyzed by SDS-PAGE and the resolved proteins were immunoblotted with anti-Dvl3 antibody. *Panel A, Dvl3 blots of region of chromatographies ≥ ~ 750 kDa*-*M*_*r*_*. *Numbers indicate the fraction. *Panel B, quantitative analysis of Dvl3-based supermolecular complexes in cells expressed either wild-type Dvl2 or S418A-Dvl2. *Dvl3 blots were quantified and results displayed. These data are representative of 2 or more independent experiments. The calculated, relative molecular weight (*M*_*r*_) positions from the calibration curve are labeled at the top. The bottom labels indicate fraction number. Arrows indicate the precise position at which calibration proteins elute from Superdex 200. Click here for file

Additional file 5 **Mutations of Dvl3 phosphorylation sites and formation of Dvl3-based punctae in response to Wnt3a. ***Live-cell images of HEK293 cells expressing either Dvl3 or Y17D-Dvl3 or S407A-Dvl3 or S407D-Dvl3 in the absence or the presence of Wnt3a. *HEK293 cells were co-transfected with Rfz1 and either wild-type or mutant of GFP- and HA-tagged mouse Dvl3 (Y17D-Dvl3, S407A-Dvl3 and S407D-Dvl3). One day post transfection, cells were treated either without or with Wnt3a. Live cell images were captured every minute in the time-course using a confocal laser scanning microscope Olympus FluoView 1000 and commercial software. The results shown are representative of two or more independent experiments. Click here for file

Additional file 6 **Axin is essential to assembly of Dvl3-based supermolecular complexes: immunoblot data. ***Panel A*, *knockdown of Axin attenuates assembly of Dvl3-based supermolecular complexes in response to Wnt3a*. F9 cells were transfected with siRNA targeting Axin one day before transfection with Rfz1. Twenty four hr later, cells were either unstimulated or stimulated with Wnt3a for 30 min. Cell lysates then were prepared and applied to Sephacryl S-400 gel filtration column chromatography. Complexes were analyzed by SDS-PAGE and immunoblotted with anti-Dvl3 antibody. Blots are shown in the region ≥ 1.5 MDa-*M*_*r*_. Labels at top indicate the fraction number. *Panel B, expression of M3Axin mutant attenuates assembly of Dvl3-based supermolecular complexes. *F9 cells were co-transfected with Rfz1 and either wild-type Axin or M3 Axin mutant. Two days post transfection, F9 cells were either unstimulated or stimulated with Wnt3a for 30 min. Cells lysates were applied to Sephacryl S-400 gel column. Fractions were analyzed by SDS-PAGE. Resolved proteins were immunoblotted with anti-Dvl3 antibody. Blots are shown in the region ≥ 1.5 MDa-*M*_*r*_*. *Labels at top indicate the fraction number. *Panel C*, *expression of wild-type Axin rescues formation of Dvl3-based supermolecular complexes in Axin-deficient cells, whereas expression of M3 Axin mutant does not. *F9 cells were transfected with siRNA targeting Axin one day before co-transfection with Rfz1 and either wild-type Axin or M3 Axin mutant. At 24 hr post transfection, cells were either unstimulated or stimulated with Wnt3a for 30 min. Cell extracts then were prepared and subjected to chromatography on Sephacryl S-400. Fractions were analyzed by SDS-PAGE, immunoblotting, and staining with anti-Dvl3 antibody. Blots are shown in the region ≥ 1.5 MDa-*M*_*r*_*. *Labels at top indicate the fraction number. Representative blots of at least 2 separate experiments are displayed. Click here for file

Additional file 7 **Expression of Axin M3 mutant precludes assembly of very large Dvl3-based supermolecular complexes in response to Wnt3a: SEC analysis on Superdex 200. Control cells**, control F9 cell SEC chromatogram. **Axin-deficient cells, **k*nockdown of Axin attenuates assembly of Dvl3-based supermolecular complexes in response to Wnt3a. *F9 cells were transfected with siRNA targeting Axin one day before transfection with Rfz1. Next day, cells were either unstimulated or stimulated with Wnt3a for 30 min. **Axin expression**, *expression of *w*ild-type Axin alone stimulates assembly of Dvl3-based supermolecular complexes. *F9 cells were co-transfected with Rfz1 and expression vectors harboring human wild-type Axin. Two days post transfection, F9 cells were treated either without or with Wnt3a for 30 min. **Polymerization-defective Axin expression**, *expression of M3 Axin mutant blocks assembly of Dvl3-based supermolecular complexes in response to Wnt3a. *F9 cells were co-transfected with Rfz1 and expression vectors harboring human M3 Axin mutant. Two days post transfection, F9 cells were treated either without or with Wnt3a for 30 min. **Rescue of Axin-depletion by expression of wild-type Axin**, *expression of *w*ild-type Axin rescues the inability of Axin-deficient cells to assemble Dvl3-based supermolecular complexes in response to Wnt3a. *Cells were treated with siRNA targeting Axin one day before subsequent co-transfection with Rfz1 and human wild-type Axin for an additional day. Twenty four hr after the final transfection, cells were either unstimulated or stimulated with Wnt3a for 30 min. **Axin rescue by polymerization-defective Axin**, *expression of M3 Axin fails to rescue the inability of Axin-deficient cells to assembly of Dvl3-based supermolecular complexes in response to Wnt3a. *Cells were treated with siRNA targeting Axin one day before subsequent co-transfection with Rfz1 and M3 Axin mutant for an additional day. Twenty four hr after this final transfection, cells were either unstimulated or stimulated with Wnt3a for 30 min. For all experiments, cell lysates were subjected to Superdex 200 gel filtration column chromatography (SEC) and resolved fractions were analyzed by SDS-PAGE, immunoblotting and staining with anti-Dvl3 antibody. Dvl3 blots were subjected to quantification by calibrated scanning. Results displayed are representative of at least 2 independent experiments, each yielding quantitatively similar results. The calculated, relative molecular weight (*M*_*r*_) positions from the calibration curve are labeled at the top of each SEC chromatogram. The bottom labels indicate fraction number. Arrows indicate the precise position at which calibration proteins. Click here for file
