# Supplementary material for: Assembly of Dishevelled 3-based supermolecular complexes via phosphorylation and Axin
Source: J Mol Signal. 2012 Jun 29;7:8. doi: 10.1186/1750-2187-7-8 (PMC3542119; doi:10.1186/1750-2187-7-8)
Supplement: Additional file 1 — Predicted sites of phosphorylation catalyzed by CK1δ on Dvl3. Panel A, Putative phosphorylation sites by CK1δ. Mouse Dvl3 domains are shown: DIX domain, yellow; PDZ domain, pink; DEP domain, blue. Putative phosphorylation sites are shown by bold character. Red characters display mutation employed in this study. Panel B, Alignment of Dsh and mouse Dvls. Sequences displayed are those flanking predicted phosphorylation sites of interest on Dvl3. Conservation of residues, corresponding to residues Y17 and S407 of Dvl3 are projected onto Dsh and Dvl isoforms, blocked written squares. [file 1750-2187-7-8-S1.pdf]

A

mouse Dvl3

```
1   MGETKIIYHL DGQETPYLVK LPLPAERVTL ADFKGVLQRP SYKFFFKSMD DDFGVVKEEI  DIX
61  SDDNAKLPCF NGRVVSWLVS AEGSHPEPAP FCADNPSELP PSMERTGGIG DSRPPSFHPH
121 ASGGSQENLD NDTETDSLVS AQRERPRRRD GPEHAARLNG TTKGERRREP GGYDSSSTLM
181 SSELETTSFF DSDEDDSTSR FSSSTEQSSA SRLMRRHKRR RRKQKVSRIE RSSSFSSITD
241 STMSLNIITV TLNMEKYNFL GISIVGQSNE RGDGGIYIGS IMKGGAVAAD GRIEPGDMLL  PDZ
301 QVNEINFENM SNDDAVRVLR EIVHKPGPIT LTVAKCWDPS PRGCFTLPRS EPIRPIDPAA
361 WVSHTAAMTG TFPAYGMSPS LSTITSTSSS ITSSIPDTER LDDFHLSIHS DMAAIVKAMA
421 SPESGLEVRD RMWLKITIPN AFIGSDVVDW LYHNVEGFTD RREARKYASN LLKAGFIRHT  DEP
481 VNKITFSEQC YYIFGDLCGN MANLSLHDHD GSSGASDQDT LAPLPHPGAA PWPMAFPYQY
541 PPPPHPYNPH PGFPELGYSY GGGSASSQHS EGSRSSGSNR SGSDRRKEKD PKAGDSKSGG
601 SGSESDHTTR SSLRGPRERA PSERSGPAAS EHSHRSHHSL TSSLRSHHTH PSYGPPGVPP
661 LYGPPMLMMT PPPAAMGPPG APPGRDLASV PPELTASRQS FRMAMGNPSE FFVDVM
```

B

```
Dsh Dros     ---MDADRGGGQETKVIYHIDDETTPYLVKIPIPSAQVTLRDFKLVLN-KQNNNYKYFFK 56
Dvl1 mouse   ----------MAETKIIYHMDEEETPYLVKLPVAPERVTLADFKNVLSNRPVHAYKFFFK 50
Dvl2 mouse   MAGSSAGGGGVGETKVIYHLDEEETPYLVKIPVPAERITLGDFKSVLQ-RPAG-AKYFFK 58
Dvl3 mouse   ----------MGETKIIYHLDGQETPYLVKLPLPAERVTLADFKGVLQ-RPS--YKFFFK 47
                         ***:***:* : ******:*:.. ::** *** **. :     *:***

Dsh Dros     -PIKERLD------QNNLEEIVKAMTKPDSGLEIRDRMWLKITIPNAFIGADAVNWVLEN 436
Dvl1 mouse   VPGAPQLEEAPLTVKSDMSAIVRVMQLPDSGLEIRDRMWLKITIANAVIGADVVDWLYTH 457
Dvl2 mouse   LPD--GCEGRGLSVHMDMASVTKAMAAPESGLEVRDRMWLKITIPNAFLGSDVVDWLYHH 467
Dvl3 mouse   IPDTERLDDFHLSIHSDMAAIVKAMASPESGLEVRDRMWLKITIPNAFIGSDVVDWLYHN 454
              *     :      : ::  :.:.*  *:****:**********.**.:*:*.*:*:  :
```
