# Supplementary material for: Assembly of Dishevelled 3-based supermolecular complexes via phosphorylation and Axin
Source: J Mol Signal. 2012 Jun 29;7:8. doi: 10.1186/1750-2187-7-8 (PMC3542119; doi:10.1186/1750-2187-7-8)

Additional file 2, Yokoyama *et al.*

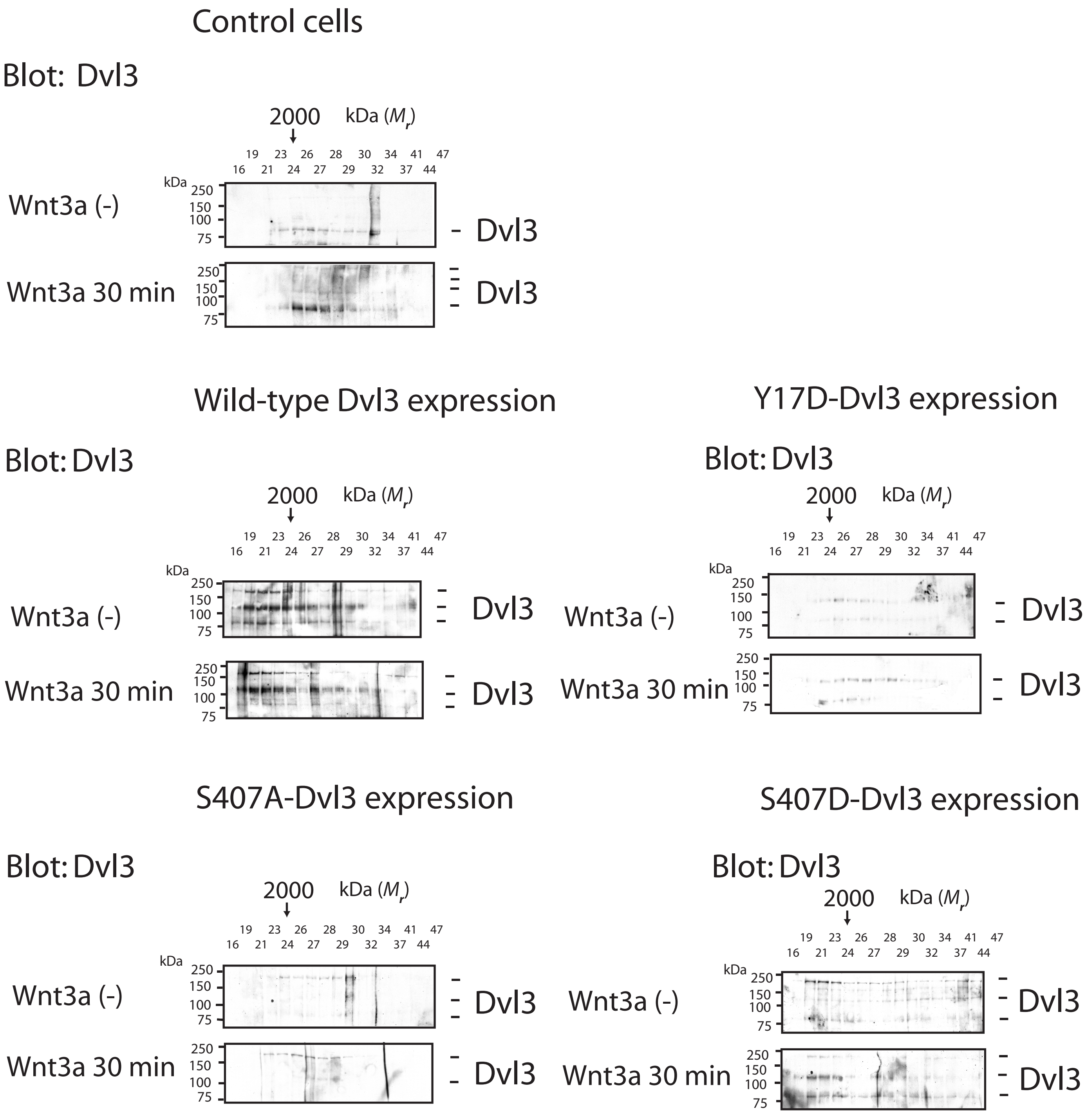

Supplement: Additional file 2 — The assembly of Dvl3-based supermolecular complexes in response to expression of Dvl3 mutants: immunoblot data. Expression of eitherY17D-Dvl3 or S407A-Dvl3 abolishes assembly of Dvl3-based supermolecular complexes, whereas expression of S407D-Dvl3 enhances assembly of Dvl3-based supermolecular complexes in response to Wnt3a. Cells expressing either wild-type Dvl3 or Y17D-Dvl3 or S407A-Dvl3 or S407D-Dvl3 were stimulated either with or without Wnt3a for 30 min. Cells were lysed and extracts (20 mg protein) were applied to Sephacryl S-400 gel filtration column (AKTA, GE Health Care). Fractions were analyzed by SDS-PAGE and immunoblotted with anti-Dvl3 antibody. Blots are shown in the region with Mr greater than1.5 MDa. Top labels specify the fraction number. [file 1750-2187-7-8-S2.pdf]
