# Supplementary figures and images for: Assembly of Dishevelled 3-based supermolecular complexes via phosphorylation and Axin
Source: J Mol Signal. 2012 Jun 29;7:8. doi: 10.1186/1750-2187-7-8 (PMC3542119; doi:10.1186/1750-2187-7-8)

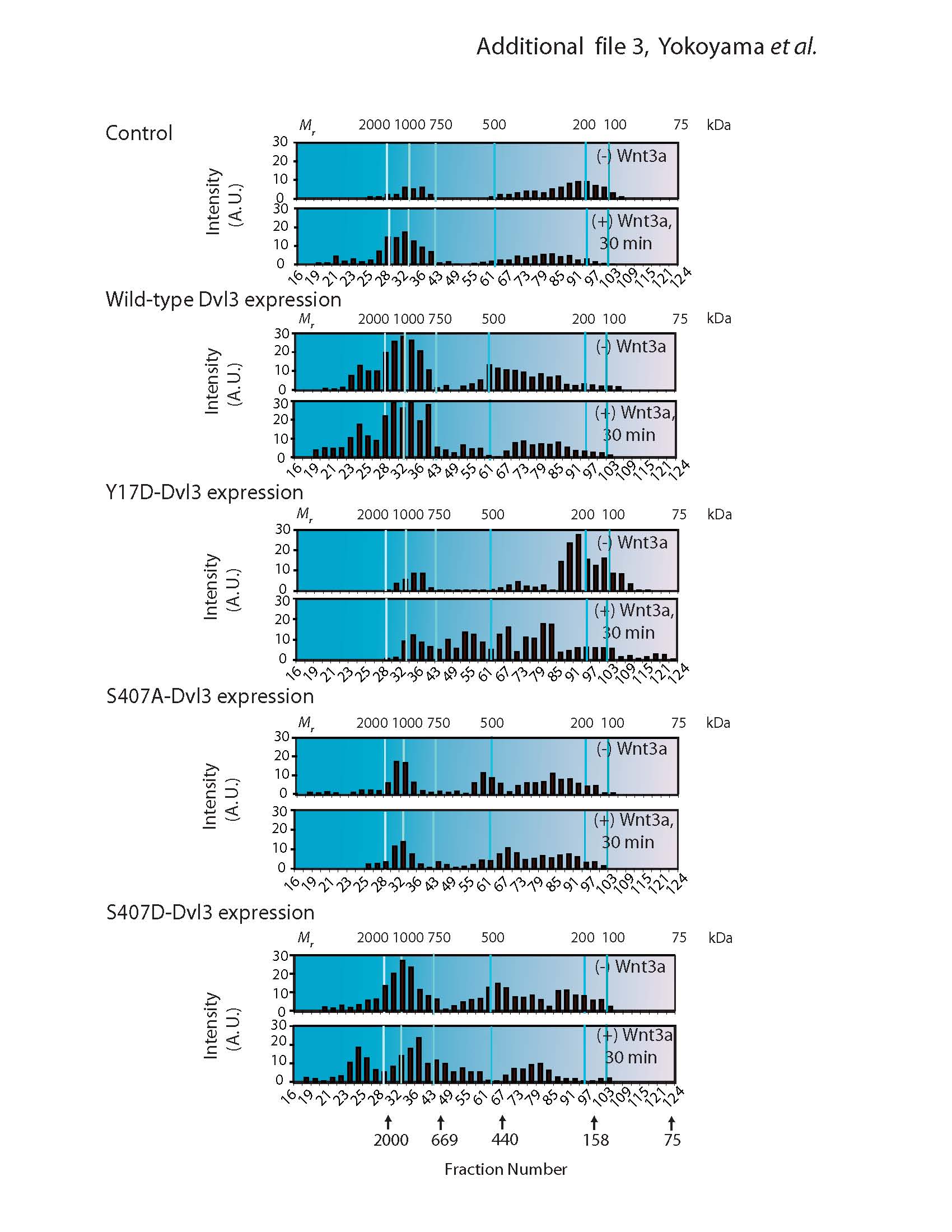

Supplement: Additional file 3 — Interrogation of Y17 and S407 sites of Dvl3 in the context assembly of lower- Mr Dvl3-based supermolecular complexes in response to Wnt3a: SEC analysis on Superdex 200. Y17D-Dvl3 and S407A-Dvl3 abolishes the assembly of Dvl3-based supermolecular complexes in response to Wnt3, whereas expression of S407D-Dvl3 enhances the assembly of Dvl3-based supermolecular complexes. Cells expressing either wild-type Dvl3 or Y17D-Dvl3 or S407A-Dvl3 or S407D-Dvl3 were stimulated either with or without Wnt3a for 30 min. Cells lysates (20 mg protein) were analyzed by SEC. The Dvl3-based complexes with Mr ≤ 1.5 MDa were characterized by Superdex 200 gel filtration column (AKTA, GE Health Care). Fractions were analyzed by SDS-PAGE and immunoblotted with anti-Dvl3 antibody. Dvl3 blots were quantified by the calibrated scanner and results were displayed. The Dvl3-based supermolecular complexes in F9 cells expressing Rfz1 were also displayed as a control. The calculated, relative molecular weight (Mr) positions from the calibration curve are labeled at the top. The bottom numbers indicate fraction number. Arrows indicate the precise position at which calibration proteins elute from Superdex 200. Results are representative of at least 2 independent experiments. [file 1750-2187-7-8-S3.jpeg]

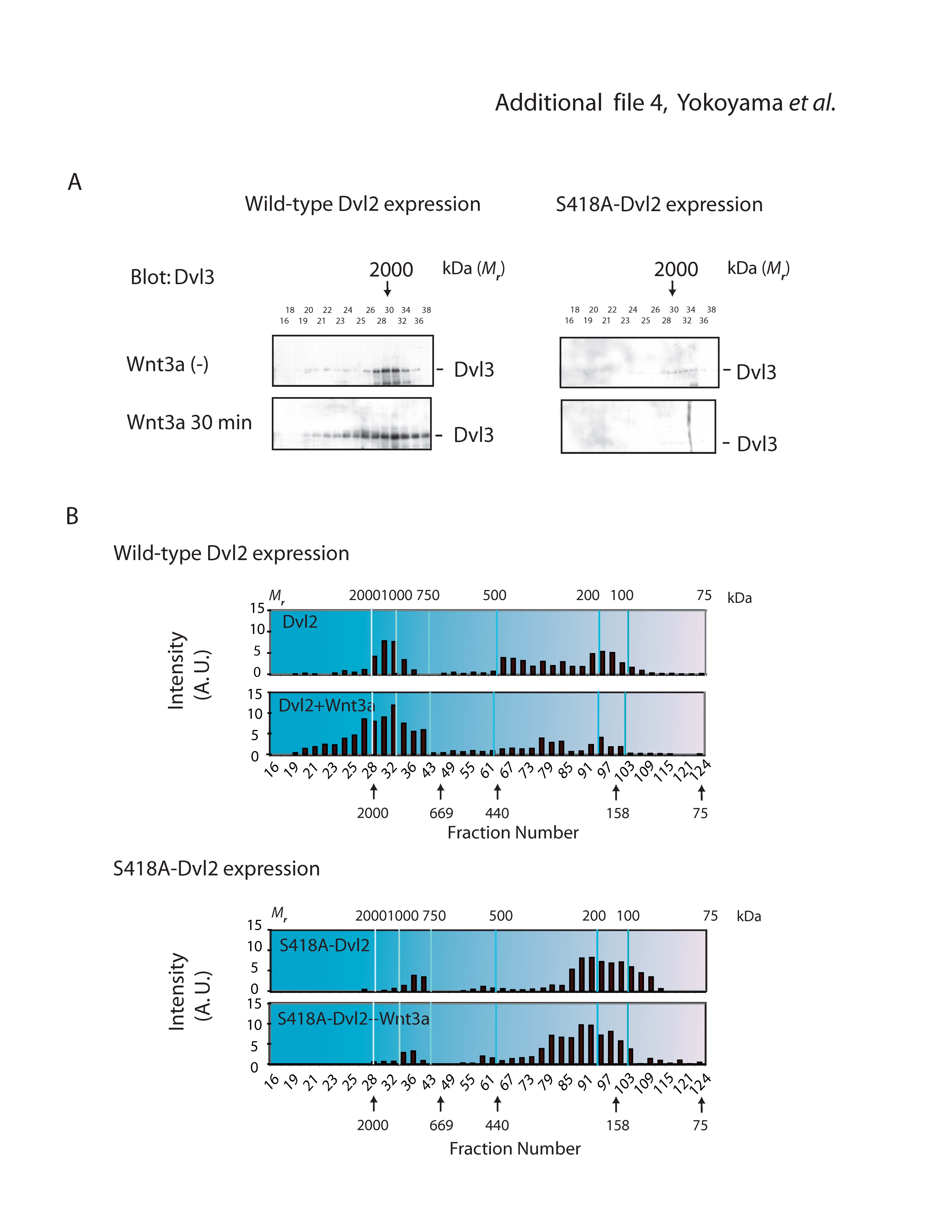

Supplement: Additional file 4 — Expression of S418A-Dvl2 abolishes the assembly of Dvl3-based supermolecular complexes. F9 cell were co-transfected with Rfz1 and either wild-type Dvl2 or S418A-Dvl2. Two days post transfection, cells were either treated or untreated with Wnt3a for 30 min. Cell lysates were subjected to characterization by SEC on Superdex 200 matrixes. Fractions separated by SEC were analyzed by SDS-PAGE and the resolved proteins were immunoblotted with anti-Dvl3 antibody. Panel A, Dvl3 blots of region of chromatographies ≥ ~ 750 kDa-Mr. Numbers indicate the fraction. Panel B, quantitative analysis of Dvl3-based supermolecular complexes in cells expressed either wild-type Dvl2 or S418A-Dvl2. Dvl3 blots were quantified and results displayed. These data are representative of 2 or more independent experiments. The calculated, relative molecular weight (Mr) positions from the calibration curve are labeled at the top. The bottom labels indicate fraction number. Arrows indicate the precise position at which calibration proteins elute from Superdex 200. [file 1750-2187-7-8-S4.jpeg]

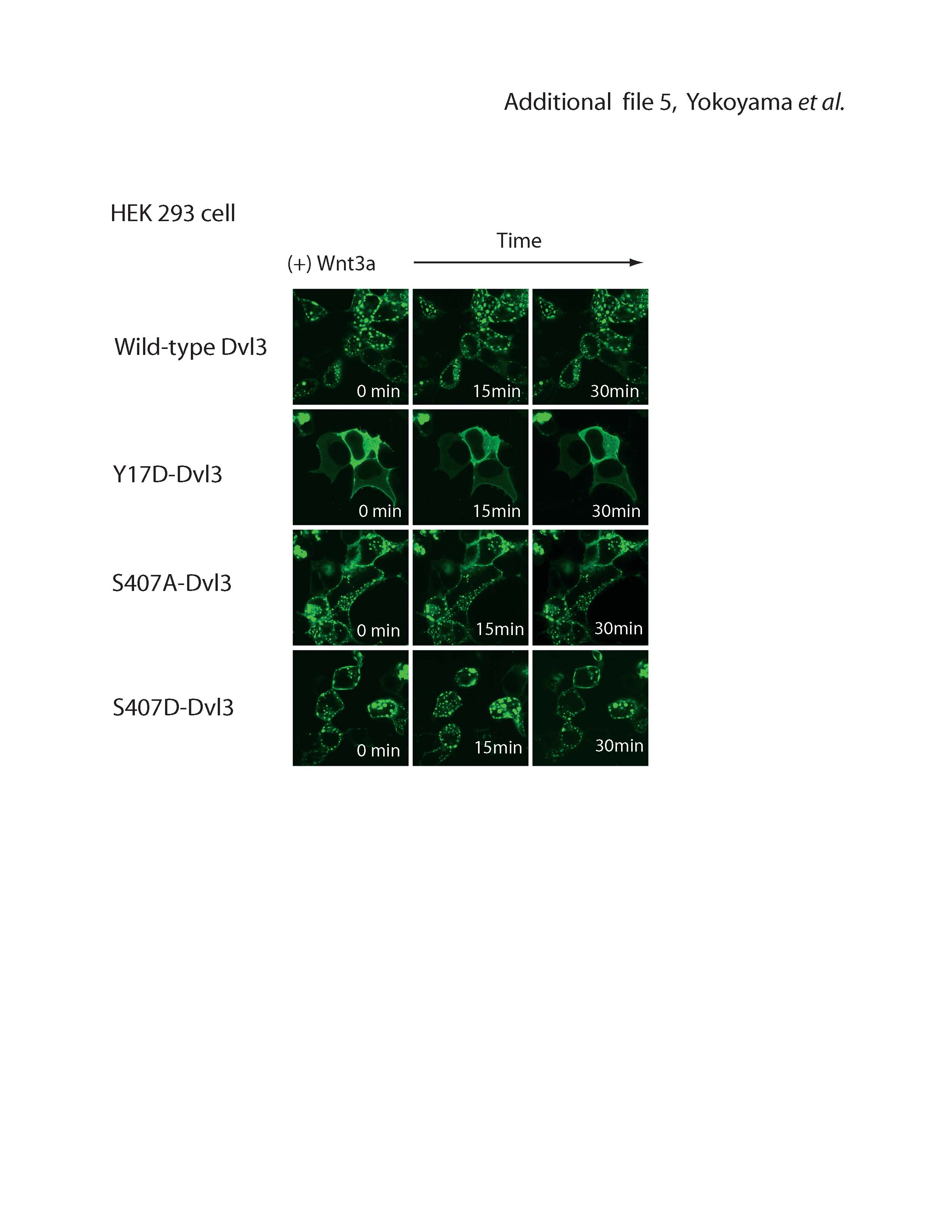

Supplement: Additional file 5 — Mutations of Dvl3 phosphorylation sites and formation of Dvl3-based punctae in response to Wnt3a. Live-cell images of HEK293 cells expressing either Dvl3 or Y17D-Dvl3 or S407A-Dvl3 or S407D-Dvl3 in the absence or the presence of Wnt3a. HEK293 cells were co-transfected with Rfz1 and either wild-type or mutant of GFP- and HA-tagged mouse Dvl3 (Y17D-Dvl3, S407A-Dvl3 and S407D-Dvl3). One day post transfection, cells were treated either without or with Wnt3a. Live cell images were captured every minute in the time-course using a confocal laser scanning microscope Olympus FluoView 1000 and commercial software. The results shown are representative of two or more independent experiments. [file 1750-2187-7-8-S5.jpeg]
