# Supplementary material for: Assembly of Dishevelled 3-based supermolecular complexes via phosphorylation and Axin
Source: J Mol Signal. 2012 Jun 29;7:8. doi: 10.1186/1750-2187-7-8 (PMC3542119; doi:10.1186/1750-2187-7-8)

Additional file 6, Yokoyama *et al.*

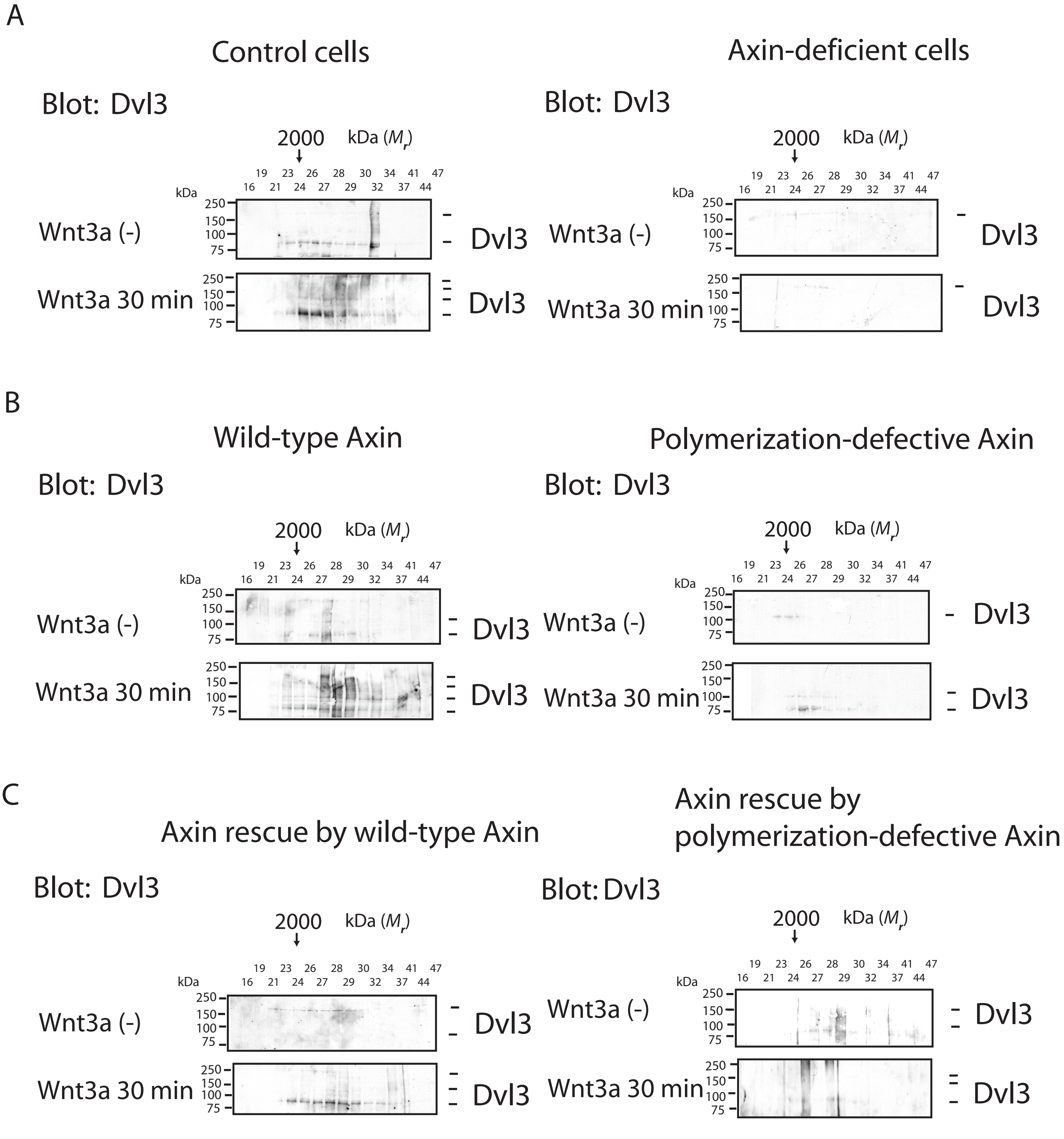

Supplement: Additional file 6 — Axin is essential to assembly of Dvl3-based supermolecular complexes: immunoblot data. Panel A, knockdown of Axin attenuates assembly of Dvl3-based supermolecular complexes in response to Wnt3a. F9 cells were transfected with siRNA targeting Axin one day before transfection with Rfz1. Twenty four hr later, cells were either unstimulated or stimulated with Wnt3a for 30 min. Cell lysates then were prepared and applied to Sephacryl S-400 gel filtration column chromatography. Complexes were analyzed by SDS-PAGE and immunoblotted with anti-Dvl3 antibody. Blots are shown in the region ≥ 1.5 MDa-Mr. Labels at top indicate the fraction number. Panel B, expression of M3Axin mutant attenuates assembly of Dvl3-based supermolecular complexes. F9 cells were co-transfected with Rfz1 and either wild-type Axin or M3 Axin mutant. Two days post transfection, F9 cells were either unstimulated or stimulated with Wnt3a for 30 min. Cells lysates were applied to Sephacryl S-400 gel column. Fractions were analyzed by SDS-PAGE. Resolved proteins were immunoblotted with anti-Dvl3 antibody. Blots are shown in the region ≥ 1.5 MDa-Mr. Labels at top indicate the fraction number. Panel C, expression of wild-type Axin rescues formation of Dvl3-based supermolecular complexes in Axin-deficient cells, whereas expression of M3 Axin mutant does not. F9 cells were transfected with siRNA targeting Axin one day before co-transfection with Rfz1 and either wild-type Axin or M3 Axin mutant. At 24 hr post transfection, cells were either unstimulated or stimulated with Wnt3a for 30 min. Cell extracts then were prepared and subjected to chromatography on Sephacryl S-400. Fractions were analyzed by SDS-PAGE, immunoblotting, and staining with anti-Dvl3 antibody. Blots are shown in the region ≥ 1.5 MDa-Mr. Labels at top indicate the fraction number. Representative blots of at least 2 separate experiments are displayed. [file 1750-2187-7-8-S6.pdf]
