# Supplementary material for: Assembly of Dishevelled 3-based supermolecular complexes via phosphorylation and Axin
Source: J Mol Signal. 2012 Jun 29;7:8. doi: 10.1186/1750-2187-7-8 (PMC3542119; doi:10.1186/1750-2187-7-8)

Additional file 7, Yokoyama *et al.*

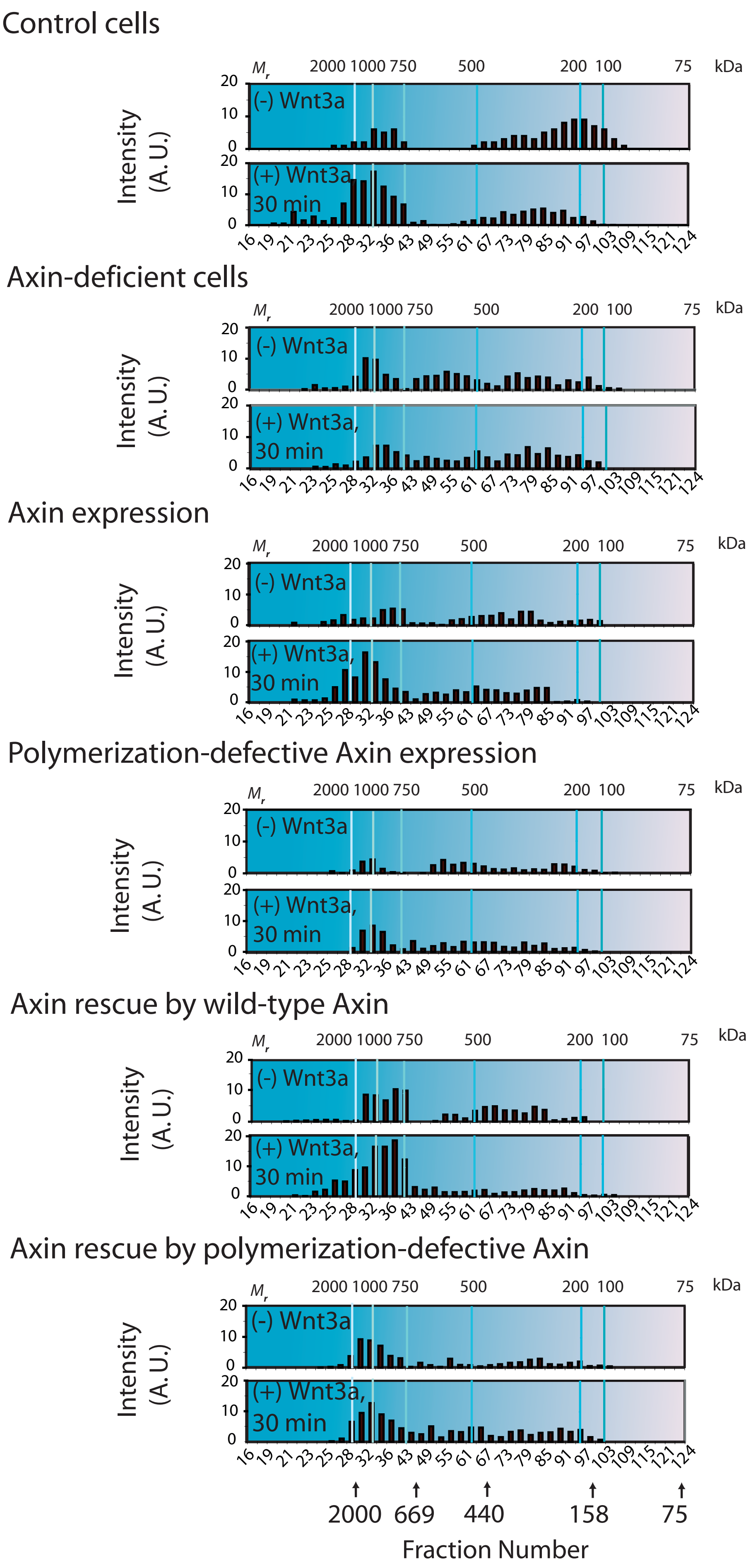

Supplement: Additional file 7 — Expression of Axin M3 mutant precludes assembly of very large Dvl3-based supermolecular complexes in response to Wnt3a: SEC analysis on Superdex 200. Control cells, control F9 cell SEC chromatogram. Axin-deficient cells, knockdown of Axin attenuates assembly of Dvl3-based supermolecular complexes in response to Wnt3a. F9 cells were transfected with siRNA targeting Axin one day before transfection with Rfz1. Next day, cells were either unstimulated or stimulated with Wnt3a for 30 min. Axin expression, expression of wild-type Axin alone stimulates assembly of Dvl3-based supermolecular complexes. F9 cells were co-transfected with Rfz1 and expression vectors harboring human wild-type Axin. Two days post transfection, F9 cells were treated either without or with Wnt3a for 30 min. Polymerization-defective Axin expression, expression of M3 Axin mutant blocks assembly of Dvl3-based supermolecular complexes in response to Wnt3a. F9 cells were co-transfected with Rfz1 and expression vectors harboring human M3 Axin mutant. Two days post transfection, F9 cells were treated either without or with Wnt3a for 30 min. Rescue of Axin-depletion by expression of wild-type Axin, expression of wild-type Axin rescues the inability of Axin-deficient cells to assemble Dvl3-based supermolecular complexes in response to Wnt3a. Cells were treated with siRNA targeting Axin one day before subsequent co-transfection with Rfz1 and human wild-type Axin for an additional day. Twenty four hr after the final transfection, cells were either unstimulated or stimulated with Wnt3a for 30 min. Axin rescue by polymerization-defective Axin, expression of M3 Axin fails to rescue the inability of Axin-deficient cells to assembly of Dvl3-based supermolecular complexes in response to Wnt3a. Cells were treated with siRNA targeting Axin one day before subsequent co-transfection with Rfz1 and M3 Axin mutant for an additional day. Twenty four hr after this final transfection, cells were either unstimulated or stimul [file 1750-2187-7-8-S7.pdf]
